# Extracellular vesicles of Fusobacterium nucleatum compromise intestinal barrier through targeting RIPK1-mediated cell death pathway

**DOI:** 10.1080/19490976.2021.1902718

**Published:** 2021-03-26

**Authors:** Le Liu, Liping Liang, Chenghai Yang, Youlian Zhou, Ye Chen

**Affiliations:** Department of Gastroenterology, State Key Laboratory of Organ Failure Research, Guangdong Provincial Key Laboratory of Gastroenterology, Nanfang Hospital, Southern Medical University, Guangzhou, China

**Keywords:** Fusobacterium nucleatum, ulcerative colitis, oncobacterium, extracellular vesicles, necroptosis, macrophages

## Abstract

Microbial factors that mediate microbes-host interaction in ulcerative colitis (UC), a chronic disease seriously affecting human health, are not fully known. The emerging oncobacterium Fusobacterium nucleatum (Fn) secretes extracellular vesicles carrying several types of harmful molecules in the intestine which can alter microbes-host interaction, especially the epithelial homeostasis in UC. However, the mechanism is not yet clear. Previously, we isolated EVs by the ultracentrifugation of Fn culture media and characterized them as the potent inducer of pro-inflammatory cytokines. Here, we examined the mechanism in detail. We found that in macrophage/Caco-2 co-cultures, FnEVs significantly promoted epithelial barrier loss and oxidative stress damage, which are related to epithelial necroptosis caused by the activation of receptor-interacting protein kinase 1 (RIPK1) and receptor-interacting protein kinase 3 (RIPK3). Furthermore, FnEVs promoted the migration of RIPK1 and RIPK3 into necrosome in Caco2 cells. Notably, these effects were reversed by TNF-α neutralizing antibody or Necrostatin-1 (Nec-1), a RIPK1 inhibitor. This suggested that FADD-RIPK1-caspase-3 signaling is involved in the process. Moreover, the observed effects were verified in the murine colitis model treated with FnEVs or by adoptive transfer of FnEVs-trained macrophages. In conclusion, we propose that RIPK1-mediated epithelial cell death promotes FnEVs-induced gut barrier disruption in UC and the findings can be used as the basis to further investigate this disease.

## 1. Introduction

Ulcerative colitis (UC), a type of inflammatory bowel disease (IBD), is chronic non-idiosyncratic inflammation of the rectum and colon mucosa. Increased disease incidence, recurrence, and prolonged disease condition can aggravate the severity, colon cancer risk, drug resistance, and toxic side effects in UC. Therefore, UC has emerged as a serious health problem of the digestive system^1^. Regrettably, the pathogenesis of UC is not yet clear. It is speculated that UC occurs due to genetic factors, psychological stress, and diet-induced adverse health effects. Also, infection and environmental factors can hyper-activate the intestinal immune and nonimmune system causing damage to the intestinal mucosal barrier.^2,3^ Moreover, altered intestinal flora and/or disorder of the immune barrier ultimately led to chronic nonspecific inflammation of the intestinal tract. Apoptosis, also known as programmed cell death (PCD), is a vital mechanism that affects intestinal mucosal injury and immune disorders in UC.^4^ PCD is triggered by the genetic program of cells and regulated by the related cytokines. The understanding of apoptosis of intestinal epithelial cells (IECs) and its related factors can elucidate the pathogenesis of UC to guide treatment for better therapeutic effects. In the last few years, UC pathogenesis has received much attention. Increasing evidence shows that apoptosis is a critical factor in UC pathogenesis^4,5^ causing superficial and extensive deletions of colonic epithelium and inflammatory changes in the lamina propria.^6^ Presently, the regulation of spontaneous apoptosis of crypt and villous axis cells is not clear; however, the involvement of Bcl-2 and Bax regulation pathway, T cell pathway, and Fas/FasL-mediated pathway^7-9^ has been investigated.

In UC, infiltrated mononuclear macrophages in the intestine synthesize inflammatory cytokines, such as TNF-α, IL-1β, and others which further trigger the cascades of inflammatory mediators. Therefore, TNF-α has been targeted for UC therapy by developing anti-TNF-α drugs. However, the role of TNF-α-dependent cellular and molecular mechanisms in UC pathogenesis is still incomprehensible. Notably, TNF-α, a multifunctional cytokine, primarily functions via tumor necrosis factor receptor R1 (TNFR1). Based on the environmental and cellular state, triggered TNFR1 induces pro-survival, pro-apoptosis, or necrotic pathways.^10^ The tumor necrosis factor receptor-related factor (TRAF2) is a crucial participant in the signal transduction pathway of TNFR1.^11^ In the TNFR1-mediated apoptosis, trimerized TNFR-1 recruits splice protein TNF receptor-associated death domain (TRADD) which converges with Fas-associated death domain (FADD), RIP, and TRAF-2 via its intracellular death domain (DD).^12^ Here, FADD binding triggers the oligomerization of caspase-8 precursor which leads to caspase cascade reaction and apoptosis. Then, interactions with RIP activate TRAF2 to form the TRADD-RIP-TRAF2 complex, which aggravates the NF-kappa B (NF-κB) signaling. The nuclear translocation of NF-κB plays an anti-apoptotic role by inhibiting the activation of caspase-8. Therefore, TNFR1 is possibly involved in two opposite signaling pathways, TRADD-FADD-caspase-3 signaling promoting cell death and pro-survival TRADD-TRAF2 signaling.^11,13^ Recent studies revealed the receptor-interacting protein kinase 1 (RIPK1) is a vital regulator of activated TNFR1 and must play an essential role in the initiation and chronicity of gut inflammation.^14^ However, the molecular details of RIPK1 mediated regulation of gut homeostasis, especially the barrier function, is largely unknown.

Fascinatingly, recent comprehensive metagenomic analysis has revealed significant microbiota variations between healthy individuals and UC patients. However, the significance of host-microbes crosstalk in the progression of UC is largely ambiguous to date. Currently, the mucosal signaling pathways, especially related to microbiota vesicles, have become the prime focus of many researchers. Bacterial vesicles can circumvent the intercellular contacts which allow the passage of critical materials such as active compounds or proteins. Extracellular vesicles (EVs) are the soluble mediators secreted by gram-negative bacteria involved in communication.^15^ Earlier research suggested a dual feature of gut bacteria EVs either being beneficiary or harmful to the host. Interestingly, the latest research has linked EVs with immunity and disease processes.^15,16^ *Fusobacterium nucleatum* (Fn), a gram-negative specific anaerobe, is involved in several diseases like preterm delivery, periodontal infection, and colorectal cancer, etc.^17,18^ Recently, it was also associated with the progression of IBD, in which it was shown to produce many pathogenic factors, such as *Fusobacterium* autotransporter protein 2 (Fap2), adhesin A (FadA), and Fusobacterial outer membrane protein A (FomA) affecting the adherence or invasion of epithelial/endothelial cells.^19–21^ In IBD patients, Fn species were found in areas of inflammation while these were absent in controls. Fn species are known to induce the expression of β-defensin and TNF-α and invasion of heterogeneous human epithelial colorectal adenocarcinoma in the Caco-2 cell lines.^18,22,23^ Also, these have been associated with increased chemotherapeutic resistance in tumors via regulation of tumor cell autophagy.^23^ Akin to other gram-negative bacteria, Fn shed EVs (FnEVs) that are composed of bacterial outer membrane and periplasmic components.^24^ However, FnEVs have not been fully characterized yet. Also, their role in the progression of UC is unknown.

This work reveals the molecular mechanism of FnEVs in the progression of UC, especially involving epithelial cell death and barrier dysfunction. Using the macrophage/Caco2 co-culture system and the DSS-induced murine colitis model, we show that FnEVs promote the differentiation of pro-inflammatory macrophages and accelerate IECs necroptosis by activating FADD-RIPK1-caspase 3 signaling, ultimately aggravating intestinal barrier damage. Our results emphasize the role of FnEVs in the progression of UC.

## 2. Methods

### 2.1. Ethics approval and consent to participate

C57BL/6 mice (8-week-old, males), purchased from the Laboratory Animal Center of Southern Medical University (Guangzhou, China), were maintained in standard specific pathogen-free conditions having free access to food and water. The animal handling protocols were approved by The Institutional Animal Care and Use Committee of Southern Medical University, China.

### 2.2. Bacterial culture and EVs isolation

Fn was cultured under anaerobic conditions (99% N2 at 37°C) until optical density (600 nm) reached 1.5 as described previously.^18^ The isolation of EVs was performed as follows. Briefly, after pelleting of bacterial cultures (10000 g for 20 min), the obtained supernatants were filtred (0.22 μm) to remove parental bacterial debris and other contaminants. The supernatant was further concentrated to 1/8 of its initial volume using 100 kDa ultrafiltration membranes (Millipore, Germany), ultracentrifuged at 4°C and 60000 g for 30 min, and washed with PBS twice to obtain the crude EVs. The purified EVs were filtered (0.22 μm) again before ultracentrifugation in a 45 Ti rotor at 150000 g at 4°C for 2 h using a sucrose density gradient, followed by endotoxin removal with a Detoxi-Gel Endotoxin Removing Column (Thermo Scientific, USA). The final pellets were resuspended in PBS and stored at −80°C.

### 2.3. Nanoparticle tracking analysis (NTA) and transmission electron microscopy

The precipitated EVs were evenly dissolved in 1 mL PBS by vortexing which was then diluted to 1 × 10^12^/L. 1 ml suspension of EVs was placed in a clean colorimetric dish to estimate the particle size distribution by Malvern Panalytical particle size meter. Isolated EVs were resuspended in PBS and examined by transmission electron microscopy.

### 2.4. Experimental groups and establishment of animal model

In each group, eight to twelve animals were randomly assigned into non-colitic, colitic, low FnEVs-treated (10 μg/day in 200 mL PBS) and high FnEVs-treated (50 μg/day in 200 mL PBS) categories, respectively. Animal intake and body weight were analyzed every day. After seven days of initiating the experiment, animals were administered with 3% (w/v) dextran sulfate sodium (DSS, 36–50 kDa, Millipore Corporation, Billerica, MA, USA) mixed with drinking water to establish colitis excluding the non-colitic group. Also, in the FnEVs-treated group, FnEVs were administered every day to each mouse by gavage. After induction of colitis, all groups received 200 mL PBS while the FnEVs-treated animals continued to receive FnEVs for another three days. After 10 days, the anesthetized animals were sacrificed for further studies. For each day, the mean disease activity index (DAI) was done following Cooper et al. score criteria.^25^

### 2.5. Isolation of PBMCs and FnEVs stimulation

The peripheral blood samples (10 mL), collected in the EDTA anticoagulant tubes, were centrifuged to obtain plasma which was mixed with Ficoll separation solution (Yeasen, Shanghai, China) to isolate peripheral blood mononuclear cells (PBMCs) using density gradient centrifugation. PBMCs were cultured in RPMI 1640-medium (GIBCO, USA) containing 10% fetal bovine serum (Gibco, Grand Island, NY, USA) and human recombinant M-CSF (25 ng/ml; Peprotech, USA) in 6-well plates (NEST Biotechnology, Wuxi, China). After 4 h induction, the non-adherent cells were removed, and the adherent cells were cultured (37°C, 5% CO_2_) for 7 days to generate macrophages with one change of fresh media at day 4. For time-dependent studies, the PBMCs-derived macrophages were treated with 1 μg/mL FnEVs after 0 h, 6 h, 12 h, 24 h, and 48 h. Subsequently, to remove the debris, the culture media were collected and centrifuged at 2000 g and 4°C for 30 min and then frozen at −80°C.

### 2.6. Cells culture

Human colonic epithelial cell line Caco-2 cells were obtained from the ATCC (Rockville, USA), and cultured in DMEM medium (GIBCO, USA) with 10% fetal bovine serum (Gibco, Grand Island, NY, USA), 4 mM glutamine, 1% nonessential amino acids, 4.5 g/L D-glucose and 110 mg/L sodium pyruvate in a humidified atmosphere at 37°C with 5% CO2. For cell signaling experiments, Caco-2 cells were treated with 1 μg/mL FnEVs with or without 50 ng/mL TNF-α (Peprotech, USA), and the cells and culture supernatants were collected after 24 h.

The Transwell system was employed for the co-culture of macrophages and Caco-2 cells. In brief, differentiated PBMCs (8.5 × 10^5^/well) were added to 6-well plates (NEST Biotechnology, Wuxi, China), and Caco-2 cells (3.75 × 10^5^/well) were seeded as a cell monolayer on a polycarbonate membrane inserted in the Transwell system (0.4 um pore size; Corning Incorporated, NY, USA). Subsequently, the PBMC derived macrophages were stimulated in a medium with or without FnEVs (1 ug/mL) in the presence or absence of neutralizing anti-human TNF-a antibody (5 μg/ml; Sigma-Aldrich) or Necrostatin-1 (Nec-1, 20uM, Sigma-Aldrich) to attenuate the specific effect of TNF-α or RIPK1, respectively. After 24 h, the cells and supernatants were harvested for further studies.

### 2.7. RT-PCR

The total RNA, extracted using the TRIzol reagent (Vazyme, Nanjing, China), was reverse transcribed using a reverse transcriptase kit (Vazyme, Nanjing, China) following the manufacturer’s protocol. The subsequent quantitative real-time PCR amplification was performed with SYBR Green Premix ExTaq (Vazyme, Nanjing, China) using the Roche LC480 System. The experiments were performed in triplicate and the change in expression levels was calculated using the 2^−ΔΔCT^ method. The gene-specific primer sequences are listed in Supplementary Table 1.

### 2.8. Western blotting

The cells were lysed in RIPA buffer (Beyotime, Jiangsu, China) with PMSF and phosphatase inhibitor cocktail (Fudebio, Hangzhou, China), and the lysates were subjected to centrifugation at 12,000 × g for 10 min. The protein concentration of the supernatants was measured with the BCA kit (Beyotime, Jiangsu, China). The samples were resolved using SDS-PAGE and then transferred onto PVDF membranes (EMD Millipore, Billerica, Massachusetts, USA). The unwanted interactions were blocked with 5% skim milk for 120 min. The blocked membranes were incubated with corresponding primary antibodies targeting TRADD, FADD, RIPK1, cCASP3, ZO-1, or GAPDH (1:1,000; Affinity, USA) overnight at 4°C, followed by secondary antibodies (1:5,000; Proteintech, China) at room temperature (RT) for 60 min. Lastly, detection was performed using the enhanced chemiluminescence detection kit (Yeasen, Shanghai, China).

### 2.9. Flow cytometry

Caco-2 cells treated with FnEVs or TNF-α or inhibitors were harvested for apoptosis assay. The cells were washed with cold PBS to dissociate into single cells and then were treated with PI and FITC Annexin V (BD Pharmingen FITC Annexin V Apoptosis Detection Kit I). After 15 min of staining, the cells were assayed by flow cytometry, and the data were analyzed using the FlowJo software version 8 (FlowJo, Ashland, OR, United States). For phenotypic identification of macrophages, cells were stained with FITC-labeled anti-CD68 mAb (BD Pharmingen, USA) versus APC-labeled anti-CD86 (BD Pharmingen, USA) or FITC-labeled anti-F4/80 (BD Pharmingen, USA) mAb versus APC-labeled anti-CD86 (BD Pharmingen, USA), respectively. The percentage of CD86+ macropahges was immediately examined by flow cytometry.

### 2.10 Co-immunoprecipitation (Co-IP) assay

For co-immunoprecipitation assay, IgG or IP antibody, suspended IP Matrix and PBS were coincubated for 1 hour at 4°C. The samples were then centrifuged and washed thrice in PBS containing the phosphatase and protease inhibitors and PMSF. The supernatants were discarded every time during washing. Subsequently, the transfected cells were lysed and transferred to the matrix before incubation at 4°C overnight. Next, the matrix was centrifuged and washed 5 times. The immunoprecipitates were mixed with SDS-PAGE sample loading buffer and boiled for 10 min at 100°C. The IP and Input proteins were detected by Western blotting.

### 2.11. Antibody protein chip detection for multiple cytokines

The macrophages released cytokines were studied using the antibody-protein chip array (RayBiotech, USA) as per the manufacturer’s protocol. Briefly, the coated protein chip membrane, previously blocked in BSA buffer solution for 30 min, was incubated with culture supernatant at RT for 4 h with slow shaking. Then, the membrane was thoroughly washed to remove nonspecific conjugates. Next, the membrane was incubated with 2 ml biotin-bound antibodies at 4°C overnight, followed by thorough rewashing. Lastly, the membrane was incubated with Cy3 bound streptavidin at RT for 1 h while shaking to ensure a uniform reaction. The fluorescent signals were detected using a laser scanner at Cy3 wavelength (Axon GenePix, Sunnyvale, CA).

### 2.12. Intracellular ROS levels assay and LDH detection

The intracellular ROS levels were determined by oxidation-sensitive fluorescent probe DCFH-DA (Beyotime, Shanghai, China), and H_2_O_2_ (500 μM) was used as a positive control. Lactate dehydrogenase (LDH) activity was tested using a commercial kit (Nanjing Jiancheng, China) following the manufacturer’s instructions.

### 2.13. Fluorescein isothiocyanate transmittance detection

Caco-2 cells, seeded on top of transwell chambers in 6-well plates, were grown to confluence. Then, freshly prepared 1 mg/ml fluorescein isothiocyanate-labeled dextran (FITC-dextran, FD4; 4kDa; Sigma, USA) solution (dissolved in HBSS) was added to the upper chamber. After 37°C incubation for 2 h, a certain volume of culture medium was collected from the lower chamber and the fluorescence (excitation 485 nm, emission 525 nm) was determined using the fluorescence enzyme labeling instrument (Thermo, USA).

### 2.14. Measurement of transepithelial resistance

Caco-2 cells, seeded on top of transwell chambers, were used to estimate transepithelial cell resistance of the intestinal epithelial barrier using the Millicell-ERS system (Millipore, USA). Two transwells with only culture medium were set as blank control, and the whole measurement process was carried out at a constant temperature. Each transwell was estimated in three different directions, thrice and the average was used to find the transepithelial electrical resistance (TEER), expressed in Ω·cm2. TEER = (measured TEER-blank pore TEER) ×effective membrane area of cell culture compartment

### 2.15. Histology and immunohistochemistry (IHC)

The paraffin-embedded colon sections were subjected to hematoxylin and eosin (H&E) as described earlier.^26^ For Alcian blue-periodic acid-Schiff (AB-PAS) stainings, the paraffin sections were dewaxed with xylene and rehydrated with different concentrations of ethanol. Then, the sections were incubated with Alcian blue solution dye for 5 min, washed under running tap water for 2 min, rinsed with distilled water, and stained with periodic acid solution for 5 min. These were treated with Schiff dye solution dye for 30 min. After washing with running tap water, hematoxylin was used for re-dying for about 1 min, followed by differentiation, back blue, and dehydration. Finally, the stained sections were covered with a cover slide. Following deparaffinization and dehydration of sections, immunohistochemical analyses were performed involving antigen retrieval and application of anti-TRADD, FADD, RIPK1, cCASP3, ZO-1, Occludin, and Claudin-1 primary antibodies (1:200, Affinity, USA). This was followed by incubation with secondary antibodies conjugated with biotin and horseradish peroxidase conjugated streptavidin (Zhongshang Goldenbridge). Post DAB staining, proteins were observed microscopically (Olympus, Japan).

### 2.16. TUNEL staining

Terminal deoxynucleotidyl transferase dUTP nick end labeling (TUNEL) assay (Vazyme, Nanjing, China) was used for in situ staining of apoptotic cells in paraffinized colon tissue sections. The cells with nuclear membrane collapse and nucleus disintegration were considered apoptosis-positive cells. Briefly, the colon tissue section (4 um thick) was placed on the slide and the cells were fixed in paraformaldehyde for 20 min, followed by three times washing in PBS, each for 10 min. Then, 50 ul of TUNEL detection solution was incubated with sample at RT for 60 min, followed by thrice washing in PBS. The cell nuclei were stained with DAPI (Sigma, USA). Finally, the samples, sealed in anti-fluorescence quenching film, were observed under a fluorescence microscope (Olympus, Japan).

### 2.17. Ex vivo *and* in vivo *imaging*

The mice in each group were gavage administered with ERFP-Escherichia coli (0.2 mL, 10^8^ CFU/ml bacteria). After 24 h, the mice were sacrificed to harvest intestinal and extraintestinal organs (gut, liver, heart, spleen, and kidney) to study with the Bruker *in vivo* fluorescence imaging system (Bruker FX Pro). For Cy7 labeling, Cy7 mono NHS ester (Cyanine 7 monosuccinimidyl ester; APExBIO) was added into the EV-PBS to 5 μM and incubated for 30 min at 37 °C. Then, the mixture was spin washed, followed by an additional wash to remove the excess dye. Cy7-labeled FnEVs (2 × 10^9^) were administered to mice. After 12 h of intragastric administration, Cy7 fluorescence in the mice was recorded as described previously.

### 2.18. Adoptive transfer of macrophages

Peritoneal macrophages (1.0 × 10^6^ cells), stimulated with LPS (100 ng/mL; Sigma-Aldrich) & IFN-γ (25 ng/mL; Peprotech, USA) or 1.0 μg/mL FnEVs for 24 h in culture conditions *in vitro*, were adoptively transferred into mice by noninvasive intravenous instillation.

### 2.19. Statistical analysis

Data from multiple assays are shown as mean ± standard deviation (SD). SPSS 20.0 and Prism 8.0 programs were used for Student’s t-test or one-way analysis of variance (ANOVA) and subsequent Dunnett’s test for post hoc analysis. *p* < .05 was considered statistically significant.

## 3. Results

### 3.1. FnEVs induce significant pro-inflammatory profiles and oxidative stress in macrophages

**Figure 1.**
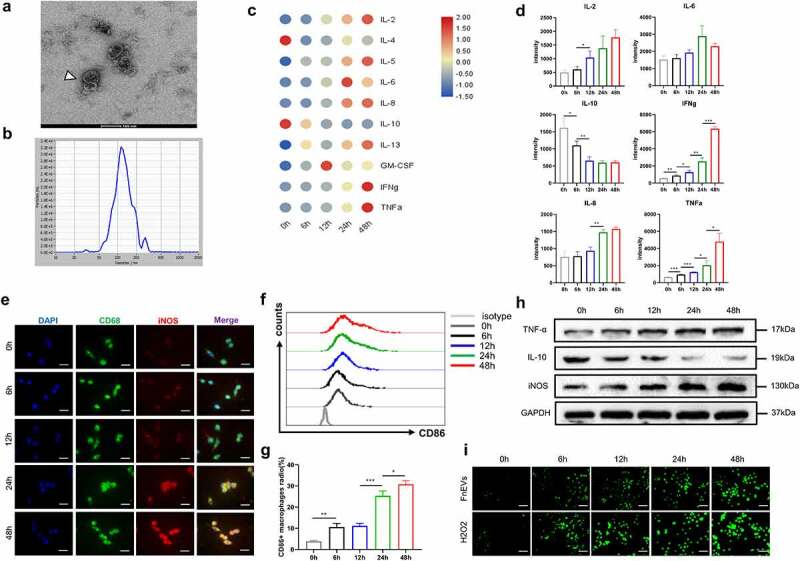
**FnEVs induce a significant pro-inflammatory profiles and ROS in macrophages**. (a)TEM of isolated FnEVs.(b) Size distribution of FnEVs analyzed by NTA. (c) Heatmap of Luminex analysis of culture supernatants of PBMC-derived macrophages stimulated with FnEVs at 0, 6, 12, 24 and 48 h. (d) Statistically significantly upregulated or downregulated cytokines measured by antibody-protein chip assay. (e) Double staining of CD68 (green) and iNOS (red). Nuclear counterstaining is provided with DAPI (blue). Scale bar = 50 um. (f) Flow cytometry analysis of M1 marker CD86 expression. (g) Bar graphs show the relative mean ratio of CD86+ cells in CD68+ population. (h) Immunoblot analysis of protein extracts from PBMC-derived macrophages with the indicated antibodies. (i) ROS was detected with a DCFH-DA staining. Scale bar = 100 um. *p < .05, **p < .01, ***p < .001. All the results were repeated three times. All data were presented as means ± SD (n = 3). PBMC, peripheral blood mononuclear cell

Bacterial EVs can be the critical mediator of IECs-microbes communication. To understand such a role in UC, we isolated FnEVs from liquid nutrient cultures using our previously established protocol. Transmission electron microscopy (TEM) was used to confirm the presence of EVs in the bacterial cultures (Figure 1a). EVs were also quantified by NTA having a specific population of 131 ± 25 nm, the peaks (n = 3) ranged from 62–310 nm (Figure 1b). To investigate the role of FnEVs primed innate macrophages in the production and secretion of pro-inflammatory cytokines, PBMCs isolated from healthy controls were stimulated with FnEVs (1.0 ug/ml), and the levels of distinct cytokines were measured in the supernatant. We observed a time-dependent increase in the expression of TNF-α and IFN-γ while the expression of anti-inflammatory IL-10 was significantly suppressed (Figure 1c-d). This prompted us to investigate the macrophage polarization, and the expressions of intestinal homeostasis regulatory factors TNF-α, IL-10, and inducible nitric oxide synthase (iNOS) in the FnEVs stimulated macrophages. The results from the double immunofluorescence (CD68 and iNOS) and flow cytometry (CD68 and CD86) experiment showed that FnEVs induced macrophage polarization into pro-inflammatory M1-like macrophages (Figure 1e-g). Furthermore, Western blotting revealed the increased levels of TNF-α and iNOS while IL-10 was suppressed in a time-dependent manner (Figure 1h). Considering the ROS involvement in macrophage polarization, we estimated the level of ROS production at different time points during macrophage activation. We found that ROS levels increased in the FnEVs-treated group with the duration of stimulation (Figure 1i). Overall, these results indicate that FnEVs promote macrophage polarization into the pro-inflammatory phenotype inducing oxidative damage in the cell.

### 3.2. FnEVs promote epithelial cells death and barrier loss in the pro-inflammatory microenvironment

**Figure 2.**
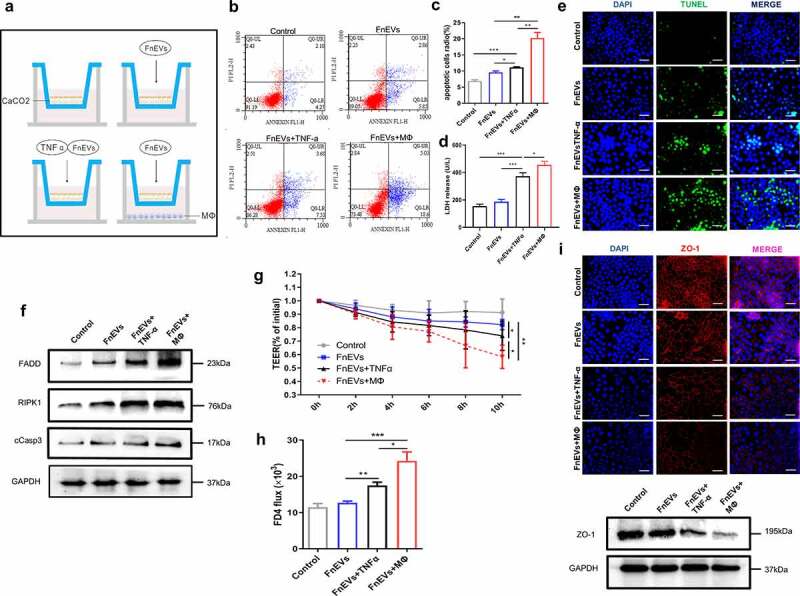
**FnEVs promote TNF-α induced Caco2 cells death and barrier loss**. (a) Diagram of groups. (b) Apoptosis was analyzed by using the annexin V FITC/PI assay. (c) Bar graphs show the relative mean of Caco2 cells apoptosis rate in different view fields. (d) The releasing levels of LDH were detected. (e) Representative images of TUNEL stainings of Caco-2 cells (green, TUNEL positive; blue, DAPI). Scale bar = 50 um. (f) Immunoblot analysis of protein extracts from Caco2 cells with the indicated antibodies. (g) Caco-2 cells were plated on a permeable membrane for TEER at different time points. (h) FITC-dextran flux was measured using Caco-2 cells grown to maximum TEER. (i) Immunofluorescence results of immunofluorescence localization of the TJ protein ZO-1(red, ZO-1 positive; blue, DAPI) and immunoblot analysis of ZO-1 protein. Scale bar = 50 um. *p < .05, **p < .01, ***p < .001. All the results were repeated three times. All data were presented as means ± SD (n = 3). TEER, transepithelial electrical resistance measurements

To determine the aggravating effect of FnEVs on epithelial cell death, we constructed macrophage/Caco2 co-cultures in transwell plates, mimicking the intestinal environment. Caco-2 cells were treated with FnEVs, FnEVs&TNF-α, or a combination of FnEVs and PBMC-derived macrophages in the transwell (Figure 2a). 24 h after the treatment, the results of Annexin-V/PI staining and TUNEL staining showed that FnEVs alone were enough to produce a subtle effect on apoptosis in Caco-2 cells. Compared with the control treatment, FnEVs&TNF-α treatment induced more severe cell apoptosis. Notably, compared to the control treatment, the combined treatment with FnEVs and macrophages remarkably increased the rate of apoptosis (Figure 2b-c,e). Lactate dehydrogenase (LDH) levels in the culture medium are the indirect indicator of cell damage. The results showed that FnEVs alone had no significant effect on LDH secretion; however, the combined treatment with FnEVs and TNF-α or macrophages significantly induced LDH release in Caco2 cell lines (Figure 2d). On the other hand, the expression levels of epithelial cell death pathway-related proteins, including FADD, RIPK1, and cCASP3, were significantly higher in the FnEVs&TNF-α group or FnEVs&macrophage group than in the control group (Figure 2f). This indicates that FnEVs may promote PCD via the TNF-α signaling pathway.

Furthermore, we also investigated the regulatory effect of FnEVs mediated-acceleration of epithelial apoptosis on barrier function by a previously established and characterized Caco-2 *in vitro* transwell model. TEER is commonly used to measure the integrity of the epithelial barrier, and a high TEER usually indicates the high integrity of the epithelial barrier. In our measurements of TEER of Caco2 monolayers, we found that treatment with FnEVs alone barely reduced the TEER values compared to the baseline after 10 h, while a combined treatment with FnEVs and TNF-α significantly decreased the TEER value. Notably, in the group of macrophage/Caco2 co-cultures with simultaneous addition of FnEVs, the decrease in TEER value was significantly higher than the FnEVs&TNF-α group (Figure 2g). Besides, the FD4 permeability assay showed that FD-4 flux changes within 24 h were consistent with that of TEER. After 24 h of treatment with FnEVs alone, FD-4 flux showed a slight increase. However, the group simultaneously treated with FnEVs and TNF-α revealed a significant change after 24 h. Besides, the FnEVs stimulation mediated increase in FD-4 of Caco-2 monolayers in the macrophage/Caco2 co-cultures was higher than in the other groups (Figure 2h). In short, the measurements of TEER and FD-4 flux across Caco-2 monolayers validated augmented epithelial permeability after incubation with FnEVs in the pro-inflammatory microenvironment. Moreover, the localization and expression of tight junctions (TJ) protein ZO-1 were evaluated in Caco-2 monolayers by immunostaining and Western blotting. The results showed that the application of FnEVs to confluent monolayers resulted in slightly diminished staining patterns of ZO-1 at the cell border, suggesting an impairment of ZO-1 protein levels. The co-treatment of cells with FnEVs and macrophages further led to a significant reduction in ZO-1 formation compared with the control group (Figure 2i). Taken together, these results suggest an important role of pro-inflammatory macrophages in the regulation of epithelial cell death and barrier dysfunction by FnEVs.

### 3.3. FnEVs induce damage to epithelial barrier via activation of RIPK1-mediated apoptosis

**Figure 3.**
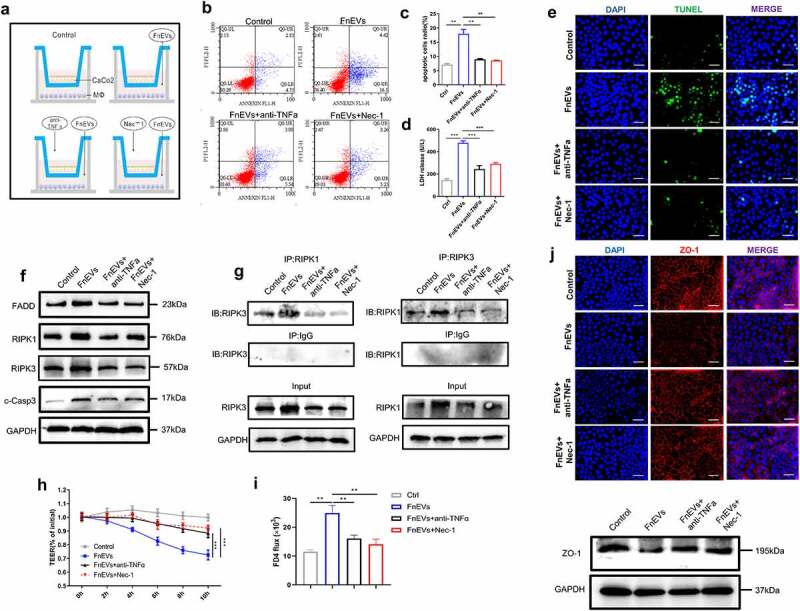
**FnEVs potentiate caco-2 cells barrier loss through RIPK1-mediated cell death pathway**. (a) Diagram of groups. (b) Apoptosis was analyzed by using the annexin V FITC/PI assay. (c) Bar graphs show the relative mean of Caco-2 cells apoptosis rate in different view fields. (d) The releasing levels of LDH were detected. (e) Representative images of TUNEL stainings of Caco-2 cells (green, TUNEL positive; blue, DAPI). Scale bar = 50 um. (f) Immunoblot analysis of protein extracts from Caco-2 cells with the indicated antibodies. (g) Immunoprecipitation of RIPK3 with its antibody caused coimmunoprecipitation of RIPK1 in Caco-2 cells. (h) Caco-2 cells were plated on a permeable membrane for TEER at different time points. (i) FITC-dextran flux was measured using Caco-2 cells grown to maximum TEER. (j) Representative images of localization of the TJ protein ZO-1(red, ZO-1 positive; blue, DAPI) stainings of Caco-2 cells and immunoblot analysis of ZO-1 protein. Scale bar = 50 um. *p < .05, **p < .01, ***p < .001. All the results were repeated three times. All data were presented as means ± SD (n = 3). TEER, transepithelial electrical resistance measurements

RIPK1 is known to participate in TNF-α receptor-mediated apoptosis.^14^ Therefore, to test the role of increased TNF-α levels or activation of RIPK1 in disruption of barrier function, we used inhibitors against them in the coculture system, namely the TNF-α neutralizing antibody or Necrostatin-1 (Nec-1, a RIPK1 inhibitor), respectively. We found that though the FnEVs treatment increased apoptosis and barrier permeability of epithelial cells in the transwell cocultures, the application of inhibitors significantly elevated the survival of Caco-2 cells, decreased FnEVs induced levels of LDH in the Caco2 cell culture supernatant (Figure 3a-e). During this process, epithelial barrier function of Caco2 cells was also significantly restored, which was indicated by increased TEER values and decreased FD4 flux of Caco-2 monolayers (Figure 3h-i). Also, immunofluorescence staining revealed that reduced epithelial distribution and expression of TJ protein ZO-1 in FnEVs-treated cells were attenuated (Figure 3j). All these strongly indicate the involvement of the RIPK1 signaling pathway in FnEVs-induced epithelial barrier dysfunction. On similar lines, Western blotting too showed that the presence of TNF-α neutralizing antibody or Nec-1 reversed the FnEVs-induced downregulation of FADD, RIPK1, and cCASP-3 in Caco-2 cells (Figure 3f). Next, using co-immunoprecipitation, we investigated the interaction between RIPK1 and RIPK3 in FnEVs-treated cells. We noticed that immunoprecipitated RIPK1 was significantly enriched in RIPK3 in the FnEVs-treated cells, compared to the control cells, which was impaired in the presence of anti-TNF-α or Nec-1 (Figure 3g). Likewise, in a reverse experimental setup, immunoprecipitated RIPK3 was enriched in RIPK1 in the FnEVs-treated cells. This too was abrogated by anti-TNF-α or Nec-1 (Figure 3j). These results strongly indicate that FnEVs not only upregulate RIPK1 and RIPK3 but also promote their aggregation to form necrosome in Caco-2 cells, eventually causing necroptosis and impairing epithelial barrier function.

### 3.4. FnEVs aggravate DSS-induced acute colitis

**Figure 4.**
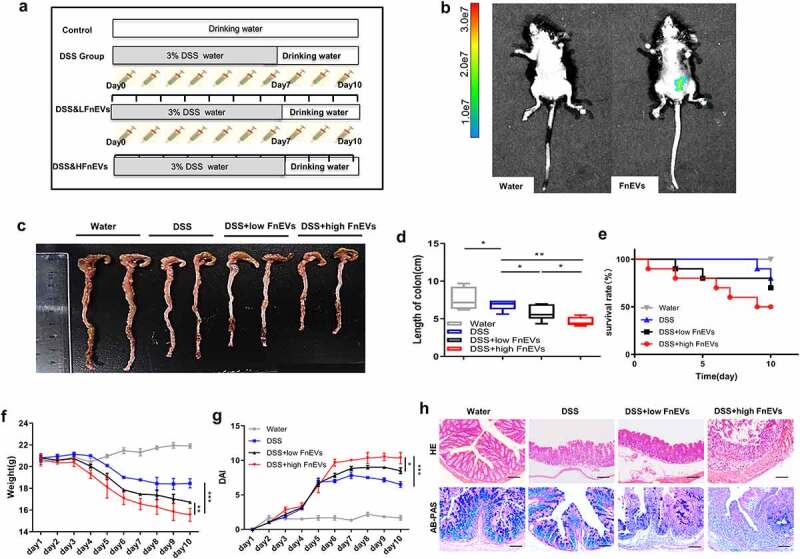
**FnEVs aggravate clinical signs of DSS-induced acute colitis**. (a) Experimental design outlining the DSS-induced colitis mice model and FnEVs treatment protocol. (b)Localization of FnEVs labeled with Cy7 at 12 h after intragastric administration. (c) Photograph of the representative colon on day 10 after colitis induction. (d) Colon length was measured on day 10 after colitis induction. (e) Effect of FnEVs on the survival rate of DSS-treated mice. (f) Effect of FnEVs on the bodyweight of 3% DSS-treated mice. (g) clinical DAI was assessed following DSS exposure. (h) H&E staining, AB-PAS staining of colon sections on day 10 after colitis induction. Scale bar = 100 um. *p < .05, **p < .01, ***p < .001. All data were presented as the means ± SD (n = 6 mice per group). DSS, dextran sulfate sodium; DAI, disease activity index. H&E, hematoxylin and eosin; AB-PAS, Alcian blue-periodic acid-Schiff

For *in vivo* study, we first used fluorescence-labeled FnEVs to verify their reach in the intestine after gavage administration. Based on the experience, Cy7-labeled FnEVs (2 × 10^9^) were administered to mice, and then after 12 h of intragastric administration, Cy7 fluorescence in the mice was acquired with the Bruker *in vivo* fluorescence imaging system (Figure 4b). We found that FnEVs reached the intestine without being significantly degraded. Then, different concentrations of FnEVs were used to intervene in mice colitis. The mice in the DSS group exhibited reduced feeding and drinking with decreased body mass, perianal dampness, and dilute stool on the 5^th^ day after the DSS challenge. Also, three mice had bloody stools on the 6^th^ day along with body mass decrease by <10%. In both DSS&low dose FnEVs and DSS&high dose FnEVs groups, almost all the mice exhibited decreased feeding, drinking, and body mass, along with lethargy, lack of grooming, diarrhea, and bloody stools on the fourth day of the modeling. On the sixth day, four mice had severe bloody stools, and three mice showed a decrease in body mass by >15%; however, no mice died in the DSS&low dose FnEVs group. On the contrary, in the DSS&high dose FnEVs group, seven mice had severe rectal bleeding, six mice lost weight >15%, and regrettably, two mice died.

In addition, DSS induced a characteristic colonic shortening compared to the control group, which was further aggravated in the DSS&FnEVs group (Figure 4c-d). The survival rate of 80.0% in the DSS group was significantly decreased in the DSS&FnEVs groups; for instance, the survival rates for 10 ug/day and 50 ug/day FnEVs-groups were 70.0% and 50.0%, respectively (Figure 4e). Also, in the DSS group, body weight was reduced after four days of DSS exposure, while the low-dose FnEVs groups showed an increase in loss of mice body weight compared to the DSS group, and the loss in body weight in the DSS&high dose FnEVs group was significantly greater than the DSS&low dose FnEVs group (Figure 4f). Similarly, the DAI score in the DSS&low dose FnEVs group was significantly higher than the DSS group and further increased in the DSS&high dose FnEVs group (Figure 4g). Histopathological analyses of intestines showed that DSS administration induced gut injuries that were not observed in the control mice. These injuries resembled those found in human UC. DSS treatment caused severe epithelial erosion, ulceration, crypt loss, and widespread infiltration of inflammatory cells in the mucosa which were further aggravated in the FnEVs treated group in a dose-dependent manner (Figure 4h). Notably, the epithelial goblet cells secrete mucins (mainly mucin2) to protect the mucus barrier and enterocytes inhibit the penetration of pathogenic microorganisms in the mucosal laminae propria. The AB-PAS staining revealed that DSS treatment led to the disruption of the mucus barrier along with a decrease in epithelial goblet cells, which were more pronounced in the FnEVs treated group in a dose-dependent manner (Figure 4h). In association with clinical parameters, these data strongly indicate that FnEVs disrupted colon histoarchitecture and increased gut damage induced by DSS administration.

### 3.5. FnEVs increase gut barrier permeability in experimental colitis model

**Figure 5.**
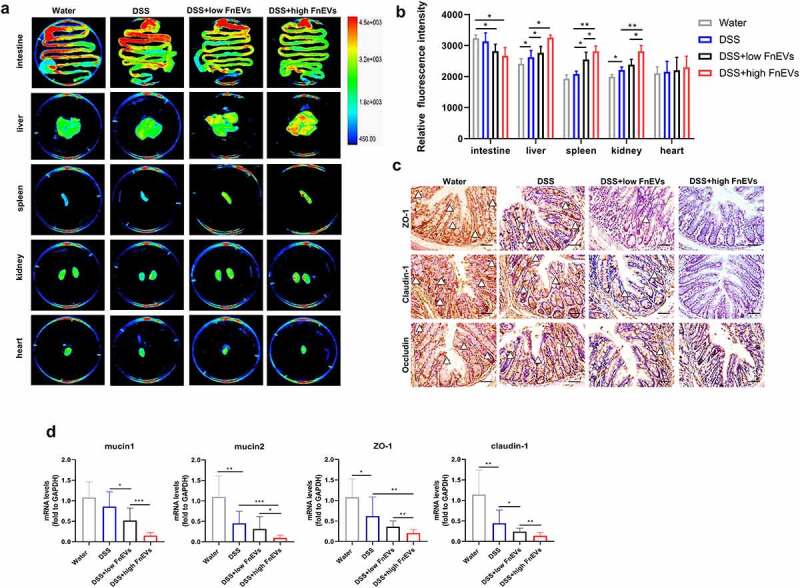
**FnEVs increase gut barrier leakage in experimental colitis models**. (a) Representative images and *ex vivo* imaging with the intestine, liver, heart, spleen and kidney of mice. (b) Relative fluorescence intensity of translocated EGFP-labeled E.coli in every tissues. (c) Representative images of immunohistochemical stainings of ZO-1, claudin-1 and occludin in the colon on day 3 after colitis induction. Scale bar = 50 um. (d) The relative mRNA level of mucin1, mucin2, ZO-1 and claudin-1 was detected in colon samples on day 3 after colitis induction. *p < .05, **p < .01, ***p < .001. All data were presented as the means ± SD (n = 6 mice per group)

Intestinal microbiota homeostasis, known to hinder the progression of UC, is critical for maintaining gut barrier integrity and epithelial restitution.^5,27^ Since there is a close association between microbes and the gut barrier function, next we evaluated the effect of FnEVs on the permeability of the intestinal barrier in inflammatory conditions. In our experiments, the mice in each group were intragastrically administered with the tracer, ERFP-E. coli (0.2 mL, 10^8^ CFU/ml bacteria) on 2^nd^ day. After 24 h, the mice were sacrificed to harvest intestinal and extra-intestinal organs (gut, liver, heart, spleen, and kidney) to study with the Bruker *in vivo* fluorescence imaging system. Our *ex vivo* imaging results revealed a dose-dependent increase in the intestinal permeability in the FnEVs treated group compared to the DSS control group. This was also indicated by an increase in translocated fluorescently labeled *Escherichia coli* in peripheral organs from the intestinal lumen (Figure 5a-b). To further evaluate the effect of FnEVs on the integrity of the intestinal barrier, we detected the levels of tight junction proteins on the third day after DSS administration with or without FnEVs treatment when the integrity of the intestinal barrier was not yet fully destroyed. Results showed that DSS treatment significantly suppressed the levels of main TJ proteins ZO-1, Claudin-1, and occludin, which was further worsened by FnEVs treatment (Figure 5c). The same phenomenon was also verified by RT-PCR in which the relative expression of mucin1, mucin2, ZO-1, and Claudin-1 was estimated in the mice colonic cells on 3^rd^ day. We noticed that in the DSS-exposed mice, the secretion of mucin1 and mucin2 was significantly reduced in the colon goblet cells which was further enhanced in the FnEVs-treated group compared to the controls (Figure 5d). FnEVs treatment also significantly decreased the levels of ZO-1 and Claudin-1 in DSS-exposed mice (Figure 5d). Thus, these results indicate that FnEVs promote epithelial barrier dysfunction, which is consistent with the exacerbated DSS-induced colitis in FnEVs-treated mice.

### 3.6. FnEVs attenuate gut barrier function via RIPK1-mediated apoptosis

**Figure 6.**
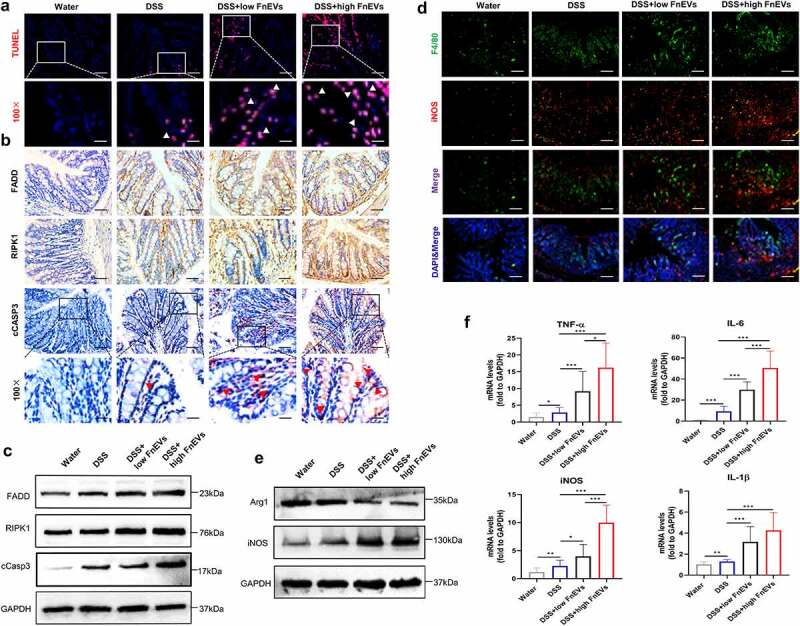
**RIPK1 mediated epithelial cell death drives exacerbated barrier loss in FnEVs-treated colitis mice**. (a) Representative images of TUNEL stainings of colon sections on day 3 after colitis induction (red, TUNEL positive; blue, DAPI). Scale bar = 50 um (up) or 20 um (down). (b) Representative images of immunohistochemical stainings of FADD, RIPK1 and cCASP3 in the colon sections on day 3 after colitis induction. Scale bar = 50 um or 20 um (downmost). (c) Immunoblot analysis of protein extracts from colon samples with the indicated antibodies. (d) Double staining of F4/80 (green) and iNOS (red) on day 3 after colitis induction. Nuclear counterstaining is provided with DAPI (blue). Scale bar = 100 um. (e) Immunoblot analysis of protein extracts from colon samples with the indicated antibodies. (f) The relative mRNA level of TNF-α, IL-6, iNOS and IL-1β was detected in colon samples on day 3 after colitis induction. *p < .05, **p < .01, ***p < .001. All data were presented as the means ± SD (n = 6 mice per group)

The homeostasis between gut pathogens and probiotics is vital for intestinal immunity and controlled epithelial apoptosis to maintain a functionally and structurally intact mucosal barrier.^15,27^ Here, on the third day of DSS treatment, we observed an increase in TUNEL-positive IECs in colonic sections of FnEVs-treated mice compared to the DSS-exposed colitis mice showing a somewhat dose-dependent effect (Figure 6a). Besides, caspase-3, a crucial enzyme in apoptosis, is a critical downstream regulator of the RIPK1-mediated cell death signaling pathway. Immunohistochemical staining showed that FnEVs treatment significantly induced the activation of FADD-RIPK1-cCASP-3 signaling which was consistent with the Western blotting analyses of intestinal samples (Figure 6b-c). These results further demonstrate the vital role of RIPK1 mediated death signals in FnEVs-induced intestinal barrier injury, which is also consistent with the results of previous *in vitro* cell experiments. Notably, the over-secretion of pro-inflammatory cytokines accelerates cell aging causing abnormal apoptosis. Therefore, we next investigated the effect of FnEVs on the production of inflammatory cytokines and the differentiation of intestinal macrophages in DSS-induced colitis on 3^rd^ day. As shown, there was an increase in F4/80+ iNOS+M1-like macrophages in the DSS group (Figure 6d-e), and the cytokines TNF-α, IL-6, IL-1β, and iNOS were also upregulated in the local colon microenvironment (Figure 6f). Interestingly, this was further aggravated in the FnEVs-treated mice compared to the controls (Figure 6d-f). Taken together, these results demonstrate that FnEVs treatment enhances IECs apoptosis by in milieu induction of pro-inflammatory macrophages causing potentially weakening of the intestinal barrier in UC.

### 3.7. Adoptive transfer of FnEVs-pretreated macrophages promoted apoptosis of intestinal epithelia

**Figure 7.**
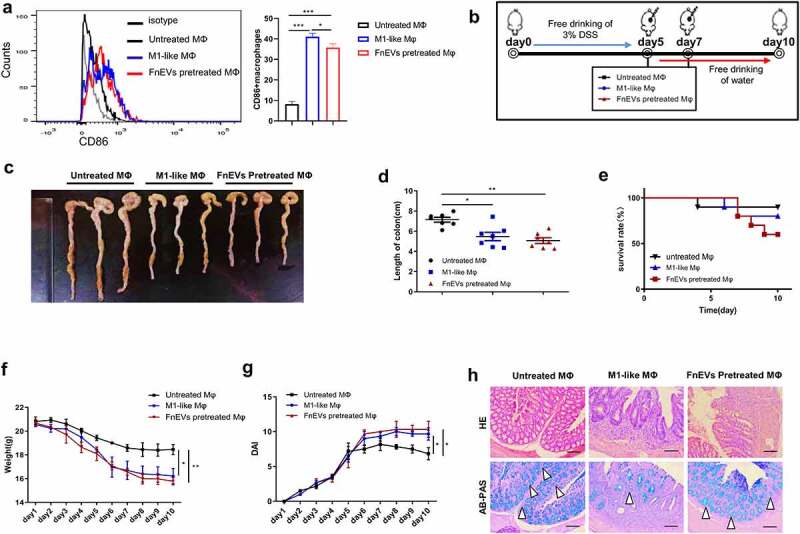
**Adoptive transfer of FnEVs-pretreated macrophages promoted intestinal epithelial death**. (a) Flow cytometry analysis of M1 marker CD86 expression and bar graphs show the relative mean ratio of CD86+ cells in F4/80+ population. (b) Experimental design outlining DSS-induced colitis mice model and adoptive transfer assay protocol. (c) Photograph of the representative colon on day 10 after colitis induction. (d) Colon length was measured on day 10 after administration of 3% DSS. (e) Effect of FnEVs-pretreated macrophages on the survival rate of DSS-treated mice. (f) Effect of FnEVs-pretreated macrophages on the bodyweight of 3% DSS-treated mice. (g) clinical DAI was assessed following DSS exposure. (h) H&E staining and AB-PAS staining of colon sections on day 10. Scale bar = 100 um. *p < .05, **p < .01, ***p < .001. All data were presented as the means ± SD (n = 6 mice per group)

To confirm that the epithelial destructive effects of FnEVs are indeed mediated by M1 macrophages, mouse peritoneal macrophages were isolated from untreated C57BL/6 mice. These macrophages were *ex vivo* treated with LPS&IFN-γ or FnEVs for 48 h to induce M1-like phenotype differentiation, which was characterized by high CD86 expression (Figure 7a). Then we performed adoptive transfer of peritoneal macrophages (1.0 × 10^6^ cells) either treated with LPS&IFN-γ, FnEVs, or vehicle through the tail vein to mice that were challenged with five-day-DSS induction. We set up LPS&IFN-γ-induced M1-like macrophage as the positive control group and FnEVs-induced M1-like macrophages as the treatment group (Figure 7b). On the 5^th^ day of the model construction, the mice in each group began to develop obvious clinical symptoms of UC (Figure 7f-g). Therefore, on the 5^th^ and 7^th^ day of the experimental process, untreated macrophages, LPS&IFN-γ treated macrophages, or FnEVs treated macrophages were transferred into colitis mice. On 10^th^ day, we found that the DSS group had less inflammation and crypt damage, epithelial defect and goblet cells also recovered substantially (Figure 7h). While the mice administered with classic LPS&IFN-γ treated M1 macrophages or FnEVs-treated macrophages began to show more severe symptoms of colitis than the untreated mice, in particular, weight loss, colon shortening, infiltration of inflammatory cells, epithelial defect, and goblet cells reduction were most common symptoms (Figure 7c-h). These results further demonstrate that FnEVs-induced pro-inflammatory phenotype transformation of gut macrophages drives progression and increases the severity of DSS-induced colitis. Also, macrophages are important cellular mediators of FnEVs mediating the apoptosis of epithelial cells in mice with colitis.

## 4. Discussion

Fn, with pathogenic traits like adhesion and invasion, has been increasingly associated with several diseases, especially those involving the inflammatory processes. Notably, Fn link with gastrointestinal disorders, especially colorectal cancer, has been explored and discussed over the past decade. However, its role in IBD has rarely been proposed. Understanding the pathogen-host interactions, which regulate inflammation-mediated damage to the host tissues, is needed to efficiently manage IBD. During bacterial infections, EVs play essential roles in intercellular communication. Studies showed that almost all gram-negative bacteria secrete EVs to activate target cells by transfer of functional molecules.^15,28^ Depending on the target cell types, bacterial species, and the number of EVs, the EVs-mediated bacterial-host interaction can elicit non-immunogenic, inflammatory, and/or cytotoxic reactions. Most gram-negative bacteria can activate caspase 11 without entering the cytoplasm.^29^ Also, EVs can be used as LPS carriers for endocytosis depended delivery into the host cytoplasm.^30^ Activation of caspase 11-dependent immune response is a critical host defense mechanism against gram-negative bacterial infection and septicemia. Apart from that, several bacteria-derived EVs, even in absence of any living bacteria, have demonstrated cytotoxicity for their virulence factors, such as leukotoxin, adenylate cyclase toxin, and hemolysin.^31–33^ For instance, *Pseudomonas aeruginosa* EVs induced cytotoxicity in human bronchial epithelial cells. Likewise, *Enterobacter coli* EVs are a common cause of passenger diarrhea and high infant mortality in third-world countries. These EVs, upon transfer of heat-sensitive enterotoxins to epithelial cells,^34,35^ induce potential activation of the pro-inflammatory pathways via endocytosis and flip-flop activation of membrane-resistant domains. Therefore, we speculated that FnEVs may mediate the intestinal epithelial responses, inflammation, and participate in the pathogenesis of IBD, independent of bacterial growth. To investigate this, we employed DSS-treated mice, macrophages/Caco2 cell co-cultures, and measured epithelial inflammatory responses both *in vivo* and *in vitro*. We found that stimulation with FnEVs robustly recruited macrophages in the intestine which were differentiated into pro-inflammatory phenotype. This significantly increased the apoptosis of epithelial cells, an important indicator of gut barrier dysfunction, in a RIPK1-dependent manner.

EVs include a variety of extracellular nanosized vesicles that can transfer bioactive lipids, proteins, mRNA, miRNA, or non-coding RNA, as means of intercellular communication to host cells. Remarkably, these can be potentially used for immunomodulation, tissue repair, or as biomarkers. However, EV isolation is a major challenge due to their nanoscale size and high heterogeneity. These concerns have been widely discussed in the international EV community (International Society for Extracellular Vesicles). There are thought to be two EV subtypes, namely 100–1000 nm diameter microvesicles (shed microvesicles, sMVs; membrane blebs) and 30–150 nm diameter exosome.^36^ The differential ultracentrifugation is the most widely used laboratory methodology, but other emerging alternative procedures may allow purer EV preparations with easy implementation. Here, we successfully isolated 30–150 nm EVs from Fn culture medium (Figure 1a-b), which provided an important basis for the further study of specific mechanisms mediated by FnEVs in UC pathogenesis. Many studies have suggested that autocrine cytokines move between IECs and macrophages.^37^ However, the mechanism of cytokines’ impact on apoptosis of IECs is not clear. TNF-α, one of the most critical cytokines in the mucosal microenvironment, induces apoptosis predominantly through the death receptor signaling pathway, involving TNFR1.^13,14,38^ Interestingly, the emerging opportunistic Fn has been linked to human gingival fibroblasts apoptosis and death of immune cells via aggregation.^39,40^ However, the exact mechanism is yet to be unraveled for conditions associated with UC. In this study, our *in vitro* results indicate that FnEVs treatment remarkably upregulated the pro-inflammatory or pro-apoptotic cytokines, including TNF-α, IFN-γ, and IL-6, while the anti-inflammatory mediators were reduced in the PBMCs-derived pro-inflammatory M1 macrophages (Figure 1c-h). As mentioned above, EVs transport various immunomodulatory molecules depending on the pathogen’s environmental conditions, regulating niche colonization, adhesion, the transmission of virulence factors, mitigating host immune response, and cytotoxicity. Recent studies showed that EVs isolated from Fn contained proteins that are predicted to localize to the outer membrane or periplasm.^24^ Of these, virulence factor FadA, anchored in the inner membrane with an extended outer membrane portion, plays an essential role in host cell invasion via interaction with epithelial E-cadherin and is elevated in periodontal inflammation. MORN2 proteins could also facilitate cell adhesion; however, the exact mechanism is not known. The other two identified surface proteins with YadA (Yersinia adhesin)-like domain are predicted to be bacterial virulence factors. Fap2, an autotransporter protein of Fn, enables interaction with TIGT to inhibit T-cell activation and NK cell cytotoxicity. The adhesins FadA and Fap2 are also suggested to induce inflammatory responses in early tumorigenesis.^19,20,24^ Therefore, EVs derived from Fn, an epithelial cell adhesion bacterium, may promote phenotypic polarization in macrophages to activate pro-inflammatory responses in the subcutaneous layer via these virulence factors.

To mimic the physiological conditions *in vitro*, we established a co-culture system of Caco-2 cells and macrophages to further investigate the specific function of FnEVs on epithelial integrity and permeability. We found that the apoptosis of Caco-2 cells and the damage to barrier function were significantly increased in the FnEVs-treated co-culture group, which indicated that apart from PCD of IECs, FnEVs augmented TNF-α induced PCD and increased epithelial permeability by creating a pro-apoptotic environment (Figure 2). Recent genetic and biochemical evidence suggests that upregulated RIPK1 signaling can induce caspase-independent apoptosis.^13,14,41^ RIP1 was initially discovered as an interaction partner for the first apoptosis signal receptor (Fas). The RIP1 death domain (DD) is necessary for interaction with other death receptor proteins, such as TNFR1, TRAILR1, TRAILR2, and other DD-containing adaptor proteins like TRADD and FADD.^42^ Nec-1 is a specific phosphorylated RIPK1 kinase inhibitor that blocks RIPK1-mediated cell death. To elucidate the exact mechanism of apoptosis induced by FnEVs, we employed TNF-α neutralizing antibody and Nec-1 against RIPK1 and its downstream factors. We found that both of these could inhibit the activation of RIPK1 in FnEVs treated PBMC/Caco-2 co-culture systems. Besides, increased permeability of the epithelial barrier and over-activated caspase-3 were also reversed by application of TNF-α neutralizing antibody and Nec-1 (Figure 3). This confirmed the involvement of the RIPK1-mediated caspase-3 signaling pathway in UC pathogenesis induced by microorganisms.

DSS model is widely used for its pathological similarity to human UC and has excellent repeatability. DSS, a synthetic polysaccharide of sulfuric acid, can affect the synthesis of DNA, inhibit the proliferation of epithelial cells, destroy the intestinal mucosal barrier, and alter the secondary immune factors which are the hallmarks of UC. Therefore, we employed a DSS-based murine model for *in vivo* studies.^43^ Mice were treated with 3% DSS for seven consecutive days to induce acute colitis. Then, different doses of FnEVs were administered through the oral route every day for 10 days, starting from day 1. The results clearly showed that FnEVs exacerbated DSS-induced colitis, which was characterized by shortening of the colon length, severely damaged structure of crypt and epithelium, and infiltration of inflammatory immune cells (Figure 4). Previous studies showed that Fn as oral pathogens is heavily enriched in the intestine of IBD patients and is closely related to intestinal inflammation.^17,18^ Therefore, Fn meditated secretion of EVs may be another exciting mechanism that triggers intestinal diseases. To clarify the effects of FnEVs on the intestinal epithelial barrier, we mainly focused on the early stage of inflammation, when the crypt structure was not yet fully destroyed. We noticed that an inflammatory response occurs centrally after gut injury. Meanwhile, after the DSS challenge, FnEVs treatment significantly promoted the infiltration and polarization of M1-like macrophages and the synthesis of pro-inflammatory mediators in colon tissues creating a specific inflammatory microenvironment (Figure 6d-f). This led to abnormal epithelial apoptosis, as indicated by TUNEL staining of the colon samples (Figure 6a). Previous evidence showed that epithelial apoptosis disrupts the TJ proteins in the colonic epithelial cells, exacerbating gut leakage.^4,44^ Notably, this is also consistent in our study (Figure 5). Though caspase-3 is well known to mediate apoptosis,^45^ the role of upstream regulatory molecule RIPK1 in FnEVs induced epithelial damage was unclear. Our results from *in vitro* and *in vivo* studies strongly showed that FnEVs applications significantly upregulated the expression of RIPK1 and its downstream signaling molecules. We believe it is the first study that links FnEVs to epithelial cell death and barrier loss via macrophage-derived TNF-α, and TNFR1-mediated RIPK1 activation (Figure 6b-c). Furthermore, anti-inflammatory M2-like macrophages increased the expressions of epithelial barrier-sealing TJ molecules to promote gut barrier repair, while pro-inflammatory M1-like macrophages, which infiltrate the colon in large numbers upon microbiota imbalance, promoted the internalization and degradation of barrier-sealing molecules, such as E-cadherin and claudin-4. This induced the expression of claudin-2, a pore-forming TJ molecule, ultimately leading to a leaky gut.^46^ In our results, the adoptive transfer of macrophages primed with FnEVs effectively promoted IECs apoptosis, increased intestinal pathological changes, mortality, and barrier destruction in murine collitis (Figure 7). Furthermore, this showed that epithelia-macrophage interactions are essential for the aggravation of FnEVs induced intestinal injury.

**Figure 8.**
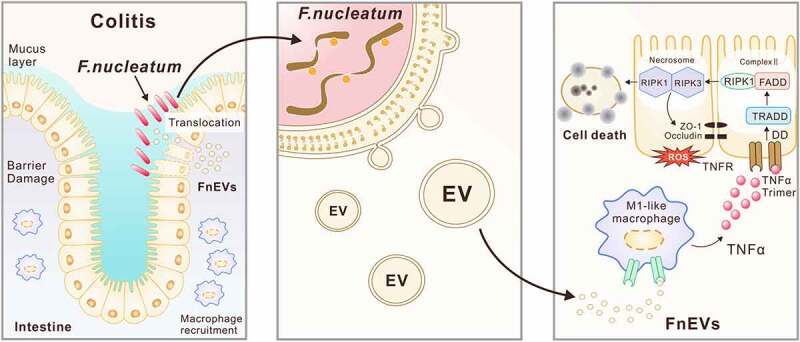
**Proposed mechanism of FnEVs-driven intestinal mucosal barrier dysfunction in UC**. Modulation of intestinal mucosal responses by Fn is thought to potentiate both Fn persistence and the development of chronic intestinal inflammation. Release of extracellular vesicles (EVs) by Fn promote macrophages to secrete proinflammatory factors that activate RIPK1 mediated cell death signals in intestinal epithelial cells, leading to the disruption of intercellular tight junctions. Thus, increased bacterial translocation induces an amplification loop of inflammation that inhibited intestinal epithelial renewal, and accelerate the progression of colitis

In conclusion, our results show that FnEVs promote disruption of the gut barrier by activating the RIPK1-mediated epithelial cell death pathway in UC (Figure 8). Therefore, targeting Fn or its secreted vesicles can promote mucosal barrier repair in UC. Most important of all, these findings may provide deeper insights into the host-microbe relationships mediated by EVs secreted from potential intestinal pathogens, and can serve as a basis for further investigations.

## Supplementary Material

Supplemental Material
